# Vaginal Hysterectomy for Malignant Disease of the Cervix, and Uterine Myoma

**Published:** 1894-04

**Authors:** L. S. McMurtry

**Affiliations:** Louisville, Ky.


ATLANTA
MEDICAL AND SURGICAL JOURNAL.

VOL. XI. APRIL, 1894. No. 2.

EDITED BY
LUTHER B. GRANDY, M.D., AND MILLER B. HUTCHINS, M.D.,
WITH THE CO-OPERATION OF
H. V. M. MILLER, M.D., LL.D., VIRGIL O. HARDON, M.D.,
FLOYD W. McRAE, M.D., ARTHUR G. HOBBS, M.D.,
AND H. R. SLACK, M.D., PH. M.

### *ORIGINAL COMMUNICATIONS.*

### VAGINAL HYSTERECTOMY FOR MALIGNANT DISEASE OF THE CERVIX, AND UTERINE MYOMA.

BY L. S. McMURTRY, M.D.,
LOUISVILLE, KY.

I wish to report two cases, which I deem of exceptional interest and illustrative of two of the most important classes of cases in pelvic surgery.

*No.* 1.—The first case is one of cancer of the uterus which I saw about ten days since in consultation with Dr. Krim. The patient had been bleeding for some time and had suffered severely with reflex nausea and vomiting. A vaginal examination revealed the unmistakable symptoms of malignant disease involving the cervix. There was also a boggy mass within the pelvis to the right of the uterus, which I feared was extension of the disease in the broad ligament, but which proved to be a pyosalpinx. One week ago yesterday I did vaginal hysterectomy and the operative technique was as follows :

For three days preparatory to the operation the patient received daily and thorough vaginal douches of a solution of bichloride of mercury. Two days prior to operation I thoroughly curetted the uterus, removing the necrosed tissues. At the time of operation the vagina was again thoroughly douched, and the cervical canal packed with iodoform gauze. These precautions are essential in order to prevent infection of the peritoneum in the course of the operation. Seizing the cervix with forceps, it was drawn well forward and the tissues divided with the scissors posteriorly, opening the peritoneum into Douglas's space. At this stage of the operation a clean sponge, to which a strong ligature was attached, was passed into the peritoneum up above the field of operation so as to prevent descent of the intestines. With the index finger of the left hand as a guide, and after drawing the cervix backward, I divided the tissues anteriorly and dissected the uterus from the bladder, being careful to avoid the ureters. The incision in the vaginal mucous membrane was completed all the way around the cervix, and the uterus brought well down. I then placed a clamp on the left broad ligament near the uterus, and attempted to treat the right broad ligament in the same way. At this period of the operation I discovered that the boggy mass, previously mentioned, was a pyosalpinx on that side. When the clamp was compressed it ruptured and pus ran down into the vagina. I divided the broad ligament on the left side, and also on the right, when I discovered that the right broad ligament was so disorganized that it would not hold the clamp. I caught up and tied the ovarian and uterine arteries on that side, and removed the diseased tube and ovary. After thoroughly irrigating, and seeing that all bleeding points were controlled, I removed the sponge from within the peritoneum, placed an iodoform gauze drain in the vagina, and around the clamps, so as to secure drainage. The patient was then placed in bed. I removed the clamps at the end of thirty-six hours; there was no hemorrhage. The patient has made an easy and uneventful recovery. She has had no vomiting since the operation, is nourishing well, and already exhibits marked improvement in her general condition.

An examination of the specimen shows that the infiltration has involved the body of the uterus, and that amputation of the cervix after any method would have failed to remove the diseased tissues. This has been the condition demonstrated in greater or less degree of every specimen that I have seen removed, and I am confident that total excision of the uterus is the only treatment worthy of consideration in cases of malignant disease of this organ. The mortality of the operation has been reduced until it is now a very safe procedure, and the ultimate results would be a great deal better if the cases could be submitted to operation earlier in the course of the disease.

### HYSTERECTOMY FOR MYOMA OF THE UTERUS.

*No.* 2.—The second case is also one of hysterectomy, but wholly different as to the character of the disease and the method of operation. The disease is a large myoma of the uterus and the operation was done by the supra-pubic route. The superior border of the tumor lay on the inferior curvature of the stomach, and occupied the entire pelvis and the greater portion of the abdomen.

An examination of the specimen shows a peculiar formation of the tumor, which very materially determined the method of dealing with the pedicle. It will be observed that the tumor has a large nodule posteriorly which filled the hollow of the sacrum, and as I open the cavity of the uterus you wili observe that the tumor is sub-peritoneal, interstitial and sub-mucous, filling up the cavity of the uterus, obliterating the cervix so as to present the form of the gravid uterus at term. The patient is thirty-two years of age, and has suffered severely from pressure and hemorrhage. She was much reduced in flesh and strength. Since the tumor impinged upon the stomach she has suffered with frequent vomiting. On opening the abdomen, the incision extending from the pubes almost to sternum, I found difficulties in dealing with the broad ligaments on each side and the bladder in front. The bladder had been carried upward by the tumor, and I had to dissect it off from the anterior surface of the growth. I ligatured the broad ligaments on each side in successive sections, dividing them as tied, until I reached the floor of the pelvis, being careful to secure the ovarian and uterine arteries. Finding the peculiar formation of the tumor which I have described, I finished the operation by complete extirpation of the cervix, and closing the vagina, being careful to suture accurately the peritoneum. I completed the operation with very little loss of blood, and put the patient in bed with a pulse of 97, without shock. I put in a glass drainage-tube. It is now seven days since the operation, and the patient has made one of the smoothest recoveries I have ever witnessed after abdominal section. I removed the drainage-tube at the end of twenty-four hours, and she has done well continuously. The pulse has remained under 100 throughout, and she has suffered no pain, never requiring a single dose of opium or of any anodyne. Indeed she received no medicine whatever except a saline purgative on the third day, and her convalescence has been exceptionally easy and uneventful. I have watched the case with unusual interest, as this is the first one of the series of abdominal hysterectomies which I have done in which I have treated the pedicle in this way. In all of my other cases I have treated the pedicle extra-peritoneally with the *neude*, and have had no reason to regret it. In this case I would have pursued that method but for the peculiar conformation of the tumor, which necessitated complete excision. This latter point will be appreciated by an examination of the specimen before you.

Until recently professional opinion made very light of fibroid tumors of the uterus. They were pronounced benign, regarded as harmless, and the menopause held up to the patient as a period when cure will come in nature's own way. With increased knowledge of pelvic diseases, it was realized that these tumors do not shrink away at the menopause, and that often they grow with increased rapidity after that period. Hemorrhage does not always cease at that period, and degenerative changes, often malignant, take place. Hemorrhage and pressure undermine the system and suffering is severe and protracted. Hypodermatic medication with ergot and local treatment by electricity may palliate, but they never cure. Surgical treatment alone can be relied upon in the treatment of these growths, a fact which is now very generally recognized by the profession.

A large proportion of fibroid tumors of the uterus may be arrested and even cured if treated in the early stages by removal of the uterine appendages. In some cases this operation will fail. It is only applicable to the early stage of these growths, and especially in cases where the hemorrhage is the conspicuous feature. In some cases where the tumor is of comparatively moderate size, one of the appendages may not be accessible by abdominal section in consequence of the altered relations made by these irregular growths. In many cases where the tumor is sub-peritoneal it may be removed by myomectomy, and the integrity of the uterus preserved. This operation is applicable to sub-peritoneal tumors which can be enucleated without opening the uterine cavity.

Hysterectomy is the operation of widest application in the treatment of uterine fibroids. Operators are divided as to the best method of treating the pedicle—one division advocating the extra-peritoneal treatment of the pedicle with the wire *neude*, and the other adopting the intra-peritoneal method. It has been objected that the *neude* is the application of the same principle in this operation as the clamp in ovariotomy, and that it should be abandoned in like manner. It must be remembered, however, that the pedicle here is composed of muscular tissue, very prone to retraction and hemorrhage, and differing in the degree of vitality from the pedicle of an ovarian cyst. The extra-peritoneal method with the *neude* is practiced by Keith, Bantock, Tait and Price; their cases are the most numerous and their results the best. This method is, as a rule, easier of execution and requires shorter time, the latter being a very important factor in avoiding shock. This method requires skill and ingenuity in securing a pedicle, reducing it to small size, so that it can occupy small space in the lower angle of the incision, and be divided by the wire without a wet slough. This method is an absolute guarantee against hemorrhage, since the pedicle is under the eye and hand of surgeon and nurse. The dropped pedicle is the method of Schrœder, and has been modified since his day. By some it is treated with several rows of sutures, and by others the cervix is completely enucleated. Results have undoubtedly improved recently by all methods, and each operator will doubtless do best with that method in which he has perfected himself and become familiar with details of managing the pedicle. In selecting between the two great methods of dealing with the pedicle it is important to bear in mind that hemorrhage is a great danger after this operation, and whatever method may be selected, the operation is a difficult one.

The results of hysterectomy for fibroid tumors have improved wonderfully within a few years past,and when the great element of time is more generaly appreciated by the profession the operative treatment will be far more successful. When the tumor is of moderate size and the system not exhausted by hemorrhage and pressure, the operation can be done with better results than in cases where the tumor has risen high up in the abdomen and the patient exhausted by loss of blood.

We have before us the report of the Society of the Lying-in Hospital of the City of New York, which affords some interesting obstetrical statistics. For the fifteen months (January, 1892, to March, 1893) there were 2,583 confinements; all but 206 were foreigners, 1,784 being Russians. The youngest patient was fifteen years of age, the oldest fifty-two. The presentation was observed in 2,328 cases. These were divided into 2,203 vertex; 80 breech; 31 shoulder; 12 face; 1 ear and 1 brow. There were 1,313 males and 1,179 females. In only 61 of the cases was it thought advisable to use ergot. The cord prolapsed in 26 out of the 2,328 cases in which the presentation was observed. Placenta previa occurred 8 times. Albuminuria occurred in 28 cases, and eclampsia in 4. Twins occurred 36 times. The forceps were applied 54 times, and version was done 64 times. Craniotomy was done twice and symphyseotomy once. Of the 2,583 cases 11 died, or one in 234.8 cases. Two of the deaths were from eclampsia; two from placenta previa, and two from rupture of the uterus.

